# Enriched Housing Enhances Recovery of Limb Placement Ability and Reduces Aggrecan-Containing Perineuronal Nets in the Rat Somatosensory Cortex after Experimental Stroke

**DOI:** 10.1371/journal.pone.0093121

**Published:** 2014-03-24

**Authors:** Alexandre Madinier, Miriana Jlenia Quattromani, Carin Sjölund, Karsten Ruscher, Tadeusz Wieloch

**Affiliations:** Laboratory for Experimental Brain Research, Division of Neurosurgery, Department of Clinical Sciences, Lund University, Lund, Sweden; Virginia Tech Carilion Research Institute, United States of America

## Abstract

Stroke causes life long disabilities where few therapeutic options are available. Using electrical and magnetic stimulation of the brain and physical rehabilitation, recovery of brain function can be enhanced even late after stroke. Animal models support this notion, and housing rodents in an enriched environment (EE) several days after experimental stroke stimulates lost brain function by multisensory mechanisms. We studied the dynamics of functional recovery of rats with a lesion to the fore and hind limb motor areas induced by photothrombosis (PT), and with subsequent housing in either standard (STD) or EE. In this model, skilled motor function is not significantly enhanced by enriched housing, while the speed of recovery of sensori-motor function substantially improves over the 9-week study period. In particular, this stroke lesion completely obliterates the fore and hind limb placing ability when visual and whisker guidance is prevented, a deficit that persists for up to 9 weeks of recovery, but that is markedly restored within 2 weeks by enriched housing. Enriched housing after stroke also leads to a significant loss of perineuronal net (PNN) immunoreactivity; detection of aggrecan protein backbone with AB1031 antibody was decreased by 13–22%, and labelling of a glycan moiety of aggrecan with Cat-315 antibody was reduced by 25–30% in the peri-infarct area and in the somatosensory cortex, respectively. The majority of these cells are parvalbumin/GABA inhibitory interneurons that are important in sensori-information processing. We conclude that damage to the fore and hind limb motor areas provides a model of loss of limb placing response without visual guidance, a deficit also seen in more than 50% of stroke patients. This loss is amenable to recovery induced by multiple sensory stimulation and correlates with a decrease in aggrecan-containing PNNs around inhibitory interneurons. Modulating the PNN structure after ischemic damage may provide new therapies enhancing tactile/proprioceptive function after stroke.

## Introduction

Stroke is the leading cause of disability in developed countries [1] and leaves 50% of the patients with motor deficits [2], and 50 to 80% of patients with loss of somatosensory functions, such as sense of limb position and touch discrimination [3]. Although lost neurological function recovers to some extent [4], 30% of stroke patients remain severely disabled through life [5]. Proprioception-based abilities such as locating the limb without looking, directing a limb to a given point, judging structure and weight of an object, are often impaired after stroke and very disabilitating [3], [6]. Transcranial magnetic stimulation, direct current stimulation, physical and cognitive rehabilitative training improve various neurological modalities even when treatment is instituted several months after stroke [7]–[9], demonstrating the innate capacity of the brain to remodel and recover some lost functions [10], [11]. This brain plasticity is considered to be the basis for spontaneous and training promoted recovery after stroke, and encompasses a set of sensori-motor and cognitive mechanisms that promote the brain to adapt to new behavioral challenges.

In the experimental setting, housing animals in an enriched environment (EE) stimulates brain plasticity by multimodal sensory, cognitive and social stimulations [12]. Enriched housing improves performance in healthy animals [13], [14] as well as in models of various central nervous system (CNS) diseases and injuries such as Huntington's disease [15], Alzheimer's disease [16], Parkinson's disease [17], fragile X syndrome [18], Down's syndrome [19] and traumatic brain injury [20].

After stroke, enriched housing improves recovery in various behavioral tests [21], [22] by stimulating brain plasticity mechanisms including synaptogenesis, growth of axons or dendrites, gliogenesis and angiogenesis [23], as well as increasing sensitivity of neurons to activation, i.e. Hebbian or homeostatic plasticity [10]. The detailed mechanisms involved in the functional recovery processes are still poorly understood, but changes in gene expression [24], [25], attenuation of the inflammatory response [26], [27], increase in dendritic branching and spine density [28], [29] have been proposed to be involved.

The extracellular matrix (ECM) is important in regulating brain plasticity [30] but is also a potential hampering factor for recovery after injury [31]–[33]. Perineuronal nets (PNNs) are highly organized, lattice-like macromolecular structures of ECM that enwrap the surface of soma and proximal dendrites of neurons [34], [35], but with distinct “holes” at sites of synaptic contacts [36]. First described by Camillo Golgi, PNNs have since then been a matter of intense research particularly in the context of neural development, axon pathfinding and guidance, plasticity and regeneration after injury (for review, see [32], [37], [38]). The main components of PNNs are hyaluronan and the lectican family of chondroitin sulfate proteoglycans (CSPGs), aggrecan, brevican, neurocan, phosphacan and versican (for review, see [39], [40]), where aggrecan is the main constituent [41]. Only approximately 15% of forebrain neurons are enwrapped by PNNs [42], [43], and these cells are mainly parvalbumin-containing GABAergic (PV/GABA) positive [44]. PNNs are found in most CNS regions, and appear to stabilize synapses [45], [46]. The number of PNNs is reduced after traumatic brain injury [47], [48], or stroke induced by middle cerebral artery occlusion (MCAO) [49], [50] and photothrombosis (PT) [51], but whether this is a detrimental or beneficial event has not been assessed.

The aim of this study was twofold: (1) to investigate how enriched housing influences brain function in a model of chronic stroke with damage to the fore and hind limb motor areas and (2) to study how enriched housing affects aggrecan-containing PNNs in the peri-infarct and somatosensory cortex after experimental stroke.

## Materials and Methods

### Permanent focal ischemia by PT

All experiments were carried out with the approval of the Malmö-Lund animal review board (ethical permit number: M 25-12) and according to the ARRIVE guidelines. Animals were housed under reverse light conditions, with the testing performed during the dark period when the rats are active. The experiments were carried out on male Sprague Dawley rats (8 weeks, Charles River). The animals were anesthetized by isoflurane (approximately 2% in O_2_ under spontaneous ventilation) and placed into a stereotaxic frame. Temperature was monitored during surgery using a rectal temperature probe and animals were kept at 37.0–37.5±0.2°C by means of a heating pad with feedback control (Table S1). A sagittal skin incision was made, subcutaneous connective tissue was removed and the skull was dried. Thereafter, the dye Rose Bengal (0.5 mL at 10 mg/mL) was injected in the tail vein. Two minutes after injection, the skull was illuminated with cold light (Schott, KL 1500 LCD) on an area of 8 to 4.5 mm for 15 min (from +4 to −4 mm antero-posterior and from 0.5 to 5 mm on the left from bregma). The tail and the scalp incisions were sutured and the rats transferred to their home cage. The functional deficit was assessed 2d after the onset of the stroke using the limb placement test and then regularly once a week.

### Randomization and EE

**Figure 1:**
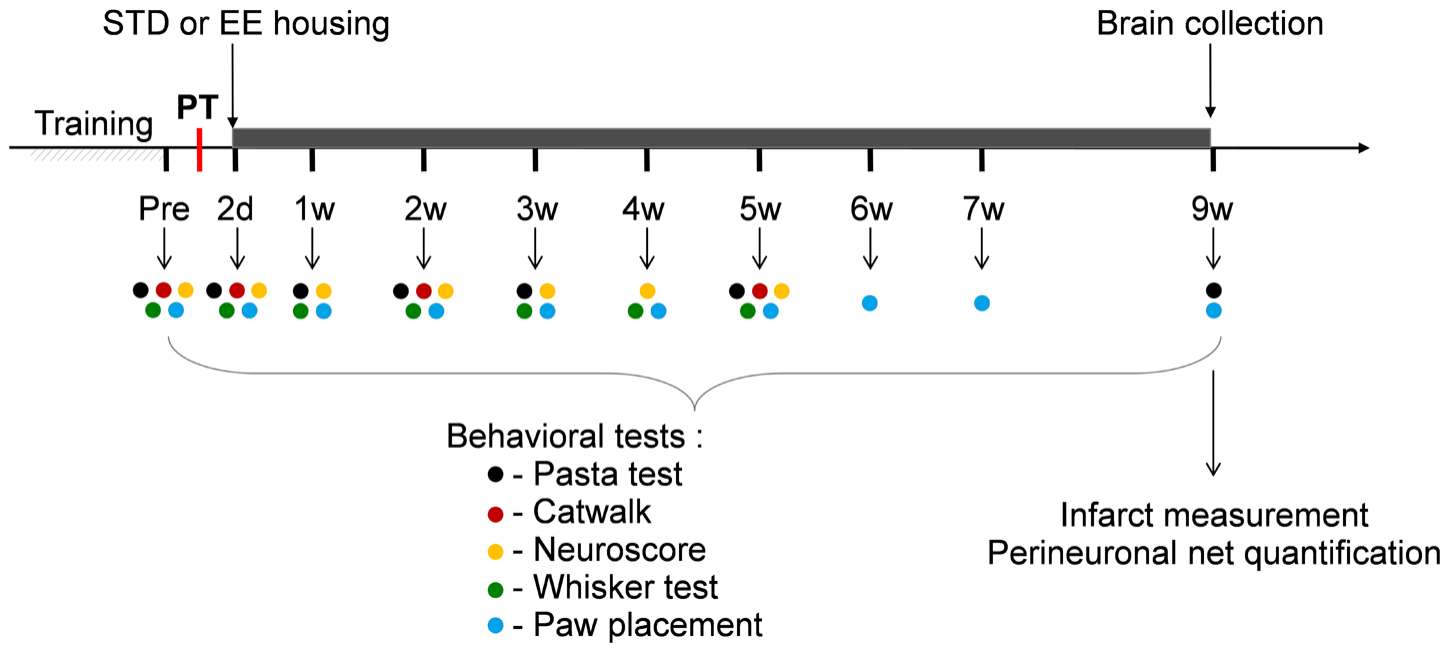
Experimental design. Rats were trained, and baseline values set for the different functional tests. Rats were then subjected to PT and the functional deficits assessed 2 days later. Subsequently, rats were placed either in standard (STD) or enriched environment (EE). Functional tests were regularly performed during 9 weeks following the onset of the stroke (the time of the different tests are represented by color dots). At 9 weeks, rats were perfusion fixed and brains were analyzed by immunohistochemistry.

On day 2 after PT, animals were randomized and kept either in pairs in standard laboratory cages (STD) or all together (7 animals) in EE cages for 9 weeks as described previously [26], [52] (Fig. 1). During the time of the experiments, the neurological function was evaluated by different behavioral tests as described below. No animal died during the surgeries and all the animals exhibited a sufficient deficit two days after stroke. In total, 14 rats were used in this study.

### Behavioral tests

#### Neuroscore

The functional deficit of the animals was assessed using a 28-point neuroscore test as described previously [53], [54]. This neuroscore uses eleven tests with a cumulative maximum score of 28: (1) circling behavior (maximum 4 points), (2) motility (maximum 3 points), (3) general condition (maximum 3 points), (4) righting reflex when placed on the back (maximum 1 point), (5) paw placement of each limb onto a table top (maximum 4 points), (6) ability to pull self up on a horizontal bar (maximum 3 points), (7) climbing on an inclined platform (maximum 3 points), (8) grip strength (maximum 2 points), (9) contralateral reflex (maximum 1 point) and (10) contralateral rotation when held by the base of tail (maximum 2 points), and (11) visual fore paw reaching (maximum 2 points). Scoring was determined on a scale starting from 0 for severe impairment to the maximum score for healthy function.

#### Paw Placement (PP)

This test provides information on the tactile/proprioceptive response to limb stimulation. Animals were placed with all 4 paws on the table top, and the paws to be tested along the edge. The rat was moved over the edge so the paws to be tested loose contact with the table surface. The ability of the animals to place the limb back onto the table surface when the rat was moved towards the edge was evaluated [55]. Importantly, the head was held at 45° angle, so that visual and whisker/snout stimulation was prevented. A prompt placement of the limb onto the table was scored as 1. Incomplete placing of the limb was scored as 0.5, and extension of the limb and paw was scored as 0.

#### Whisker paw placement test (WP)

The whisker paw placement test was performed as described in [56]. The fore limb placing assesses the ability of rats to sense tactile stimulus from the vibrissae and subsequently elicits a motor response of fore limb placing [57]. In this test, the animal's body was held gently such that the limbs were suspended off the experimenter's hand. The animal was then brought laterally to a tabletop, while the vibrissae brush against the edge of the table, allowing the animal to reach the edge with its fore paw. This was repeated 10 times on each side. The number of successful limb placements onto the edge of the table was recorded. A cross-midline variation to the test was also performed. Here, the animal was held on its side and successful placements of the opposite limb to the stimulated whisker were recorded.

#### Skilled reaching test (Pasta test)

Rats were kept under restricted feeding [58]. The aim of restricted feeding was to keep the rats 10% under the normal age/weight curve. Prior to stroke, the animals were weighted every day at the same time and, depending on their weight, they received between 22–30 g of pellet/2 rats in a cage. At 2 days after stroke, the animals received pellets (11–15 g/rat) placed at 4–5 different places in the EE cages. The STD housed rats received 11–15 g of pellet/rat, while weight was controlled three times/week. On the day before testing the amount of pellet/rat was reduced by 50%. For habituation to the pasta, four small pieces (4–5 cm long) were given to the rats in the cage, daily for 5–7 days, before initiating the training period. Animals were trained for 12 days prior to the test. For the test, the animals were placed into a Plexiglas cage with a small opening giving access to the pasta plate. The pasta plate included 10 rows of 14 holes filled with 3.5 mm long pasta pieces (2.5 cm available for grabbing). The plate was placed in front of the opening on the side of the lesion so that only pasta pieces could be reached with the paw contralateral to the lesion. The total number of pieces removed after 20 min was counted. In between tests, pasta was given twice a week into the cage.

#### Gait

Gait analysis was performed using the Catwalk automated gait analysis system (Noldus Information Technology, Wageningen, The Netherlands). Rats were trained on the walkway until 3 compliant (speed variation less than 55%, and between 15 and 115 cm.s^−1^) runs were successfully performed and recorded according to the manufacturers instructions. Thereafter, the recorded data were analyzed using the Catwalk XT 9.0 software. Each footprint was imaged and gait parameters were subsequently calculated to provide data on (1) time course of movement of each individual paw, (2) size and intensity of the contact of each individual paw, and (3) coordination (temporal and spatial) among the paws (Table S2).

### Tissue collection

Rats were deeply anesthetized (isoflurane) and perfusion-fixed with 4% paraformaldehyde (PFA) solution. Brains were collected and immersed in 4% PFA solution for 4 h, then transferred into a 25% sucrose phosphate buffer solution (0.1 M NaH_2_PO_4_, 0.1 M Na_2_HPO_4_) and stored at 4°C. Brains were then cut in 30 μm slices using a microtome and one slice was kept each 1 mm for infarct size measurement. The others were kept for immunohistochemistry and immunofluorescence. All the slices were stored in anti-freeze solution (30% ethylene glycol, 30% glycerol, 0.01 M NaH_2_PO_4_, 0.03 M Na_2_HPO_4_) at -20°C.

### Immunohistochemistry

Free-floating brain slices were rinsed three times in PBS (phosphate buffer saline) and quenched in 3% H_2_O_2_ and 10% MetOH for 15 minutes. As enhancing step for the AB1031 aggrecan staining (AB1031, Millipore), chondroitinase ABC (C3667, Sigma) digestion of the slices was first performed for 60 min at 37°C in Tris 50 mM, pH 8.0, sodium acetate 50 mM, 0.02% bovine serum albumin, containing 20 mU/mL lyase. After washing in PBS, the sections were blocked with blocking solution (5% normal donkey serum, Jackson ImmunoResearch, UK and 0.25% Triton X-100 in PBS) for one hour at room temperature. The sections were incubated overnight at 4°C with either of the following two primary antibodies: polyclonal rabbit anti-aggrecan (1∶1000, AB1031, Millipore), monoclonal mouse anti-chondroitin sulfate proteoglycan (1∶1000, protein core epitope, Cat-315/MAB1581, Millipore) diluted in blocking solution. Following three rinses with 1% normal donkey serum and 0.25% Triton X-100 in PBS, the sections were incubated with appropriate secondary biotinylated antibodies (donkey anti-rabbit/mouse, Vector Laboratories, USA) at a dilution of 1∶400 in blocking solution for 90 minutes at room temperature (RT). Visualization was achieved via the Vectorstain ABC kit (Vector) using 3,3-diaminobenzidine-tetrahydrochlorid (DabSafe, Saveen Werner, Sweden), 8% NiCl and 3% H_2_O_2_. Bright field pictures were acquired using an Olympus BX60 microscope (Solna, Sweden).

### Immunofluorescence

Free-floating brain slices were rinsed three times in PBS and digested with chondroitinase ABC (C3667, Sigma; for details, see immunohistochemistry) as enhancing step for the AB1031 aggrecan staining (AB1031, Millipore). The sections were then blocked with blocking solution (5% normal donkey serum, Jackson ImmunoResearch, UK and 0.25% Triton X-100 in PBS) for one hour at RT. The sections were incubated overnight at 4°C with a primary antibody (polyclonal rabbit anti-aggrecan 1∶1000, AB1031, Millipore; monoclonal mouse anti-chondroitin sulfate proteoglycan 1∶1000, protein core epitope, Cat-315/MAB1581, Millipore; monoclonal goat anti-parvalbumin, 1∶2500, PV235, Swant) diluted in blocking solution. Following three rinses with 2% normal donkey serum and 0.25% Triton X-100 in PBS, the sections were incubated with appropriate secondary antibodies (donkey anti-rabbit/mouse/goat antibody) conjugated with either Cy3 or DyLight 488 fluorescent dyes (Jackson ImmunoResearch, UK) at a dilution of 1∶400 in blocking solution for 90 minutes at RT. Fluorescent dyes were imaged using a confocal laser-scanning microscope (Zeiss LSM 510, Jena, Germany).

### Cell counting

Aggrecan-containing PNNs (AB1031 and Cat-315 positive) were visualized by immunohistochemistry in bright field. A 4× magnification objective was used to draw the boundaries of the regions of interest and to perform the actual cell counts by an investigator blinded to the group identity. AB1031 and Cat-315 positive PNNs were counted in the peri-infarct cortex (defined as 0.5 mm lateral from the ischemic core on the ipsilateral hemisphere and the correspondent region in the contralateral hemisphere) and in the somatosensory cortex (comprised between the peri-infarct cortex and 3.5 mm from the top of the slice on both hemispheres). Distances were defined using an optical grid. Two coronal brain sections for each animal of the study were used (level +1.20 mm in relation to bregma).

### Infarct measurement

The brain slices were stained following the immunohistochemistry protocol described above using a monoclonal mouse anti-NeuN antibody (A60/MAB377, Millipore) at the dilution of 1∶1500. All slices were scanned, the non-lesioned area of the infarcted hemisphere and the non-lesioned contralateral hemisphere were outlined and measured using ImageJ. The infarct volume was then calculated by integration of the different areas [59].

### Statistical analysis

For the analysis of PP, WP test and neuroscore, the Mann-Whitney test was used. For the analysis of skilled reaching test, the results were compared using two-factor ANOVA with post-hoc Tukeýs test. Regarding the analysis of infarct volume and cell counting, Student's t-test (unpaired) was used to compare the STD and EE groups. For the analysis of the effect of stroke on the gait (Catwalk) parameters, Student's t-test has been employed between values before stroke and values at 2 days of recovery (paired). For the analysis of differences in gait parameters between housing conditions during recovery, two-factor ANOVA was used, followed by the Student's t-test (unpaired) and Bonferroni corrections. All data are presented as mean ± SEM if not stated differently. Data and statistical processing were performed using Microsoft Excel and GraphPad Prism Software.

## Results

### Enriched housing does not affect infarct size after PT

**Figure 2:**
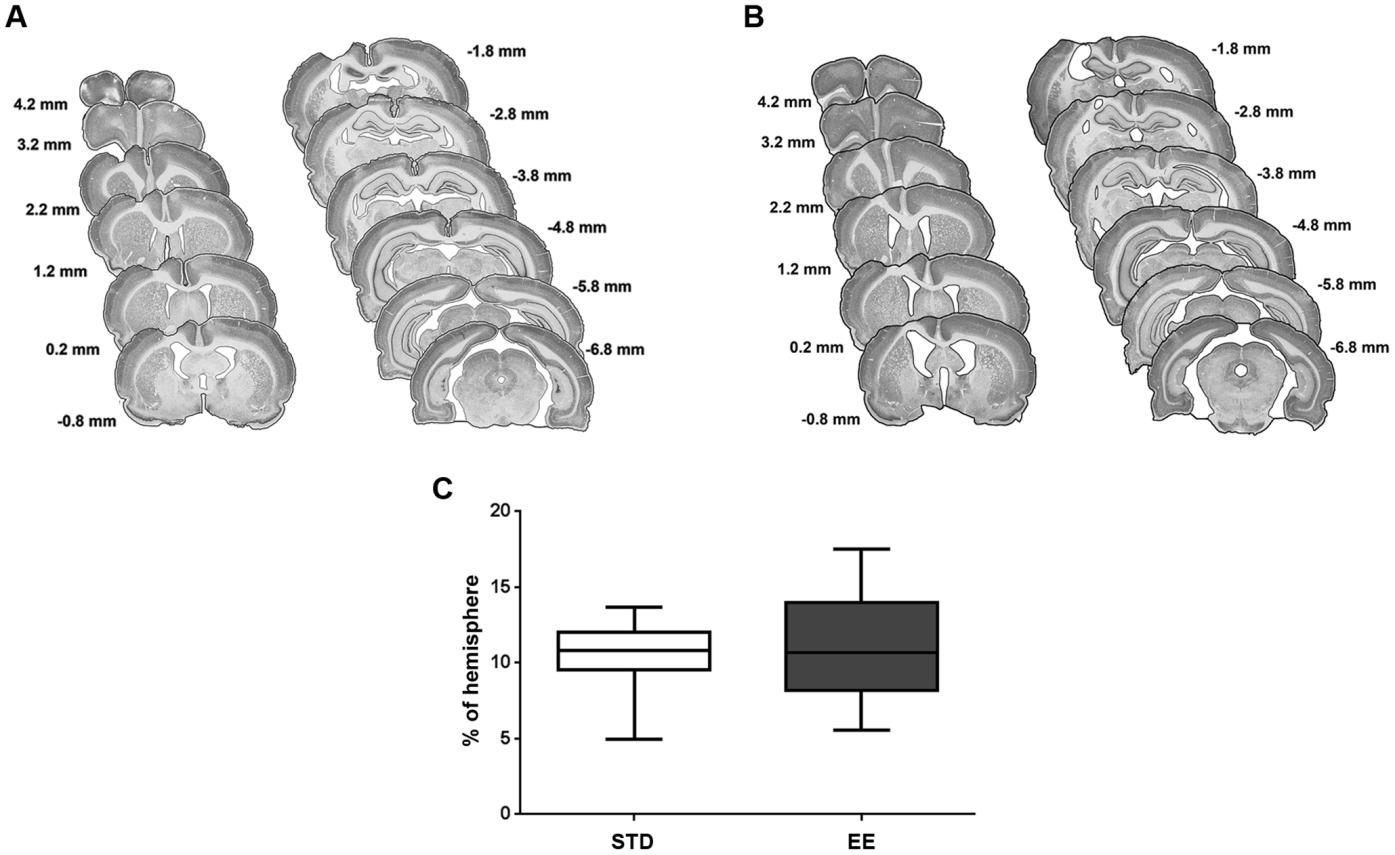
Infarct Volume. Infarct volume was measured at 9 weeks after PT, and estimated by integration of the lesioned area on brain slices taken every 1-caudal brain axis. NeuN staining was performed to discriminate between intact and infarcted tissue. (A) Successive coronal slides of a representative brain infarct with their respective distances from the bregma for the STD housed animals, and (B) EE housed animals. (C) Infarct volume expressed as percentage of the hemisphere. Values are presented in box plots: median; 1^st^ quartile to 3^rd^ quartile; minimum and maximum value. (n = 7 for each housing condition; STD: standard environment, EE: enriched environment).

Figures 2A and 2B show the location of the cortical infarct 9 weeks after induction of a PT lesion as displayed in NeuN stained coronal sections of a rat brain, along its rostro-caudal axis. The lesion extended 8 mm caudally from the frontal cortex, and included the fore and hind paw motor areas. The medial aspects, the cingulate and retrospinal cortices, were left intact, as were large parts of the somatosensory cortex. In all animals, the lesion extended from the cortical surface down to the corpus callosum, but did not transect the white matter. There was no difference in localization of the lesion between the STD (Fig. 2A) and EE (Fig. 2B) animals. Also, the mean size of the infarct in the STD animals at 9 weeks after PT stroke was 53±5 mm^3^, while in the animals housed in EE the mean infarct size was 52±7 mm^3^, and the fraction damaged cortex was the same between the groups (Fig. 2C). Hence, enriched housing starting 2 days after PT and continuing for 9 weeks did not influence the size of the infarct.

### Enriched housing does not improve skilled reaching and does not affect gait

#### Skilled reaching test

**Figure 3:**
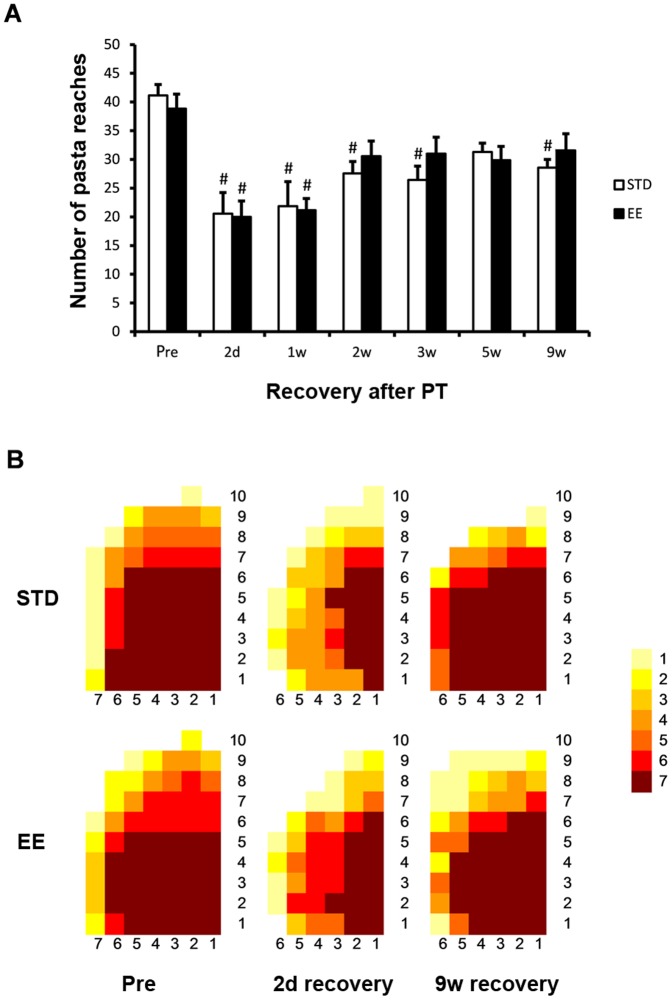
Skilled reaching test. Skilled reaching test at different times of recovery after PT stroke for animals housed in standard (STD) and enriched environment (EE). (A) The total number of pasta pieces reached during 20 minutes prior to PT (pre) and at different time points after PT. (#: p<0.05 when compared to the respective group before stroke (pre). (B) Map of the reaching area presenting the number of animals (as indicated by colors) that reached the pasta pieces (n = 7 for each housing condition).

A lesion to the motor cortex impairs paw functions in particular behavioral tests. In the pasta matrix reaching task, a test of skilled motor function [58], the number of pasta bars reached and grabbed during 20 min, before induction of stroke, was 41±6 in the STD group and 38±5 in the EE group (Fig. 3A). At 2 days after stroke, the reaching was markedly impaired and decreased by approximately 50% in both groups. At 1 week of recovery, i.e. 5 days in either STD or EE cages, the impairment remained significantly depressed and still at around 50% of the baseline values in both groups (p<0.001, Tukeýs test). Skilled motor function started to recover at 2 weeks and by 9 weeks recovered to 68% and 81% of baseline values in the STD and EE groups, respectively. The deficiency in reaching was mainly seen in the ability to reach the most distant rows of pasta pieces (Fig. 3B). Importantly, there was no significant difference between STD and EE groups at any time point of recovery.

#### Gait

**Figure 4:**
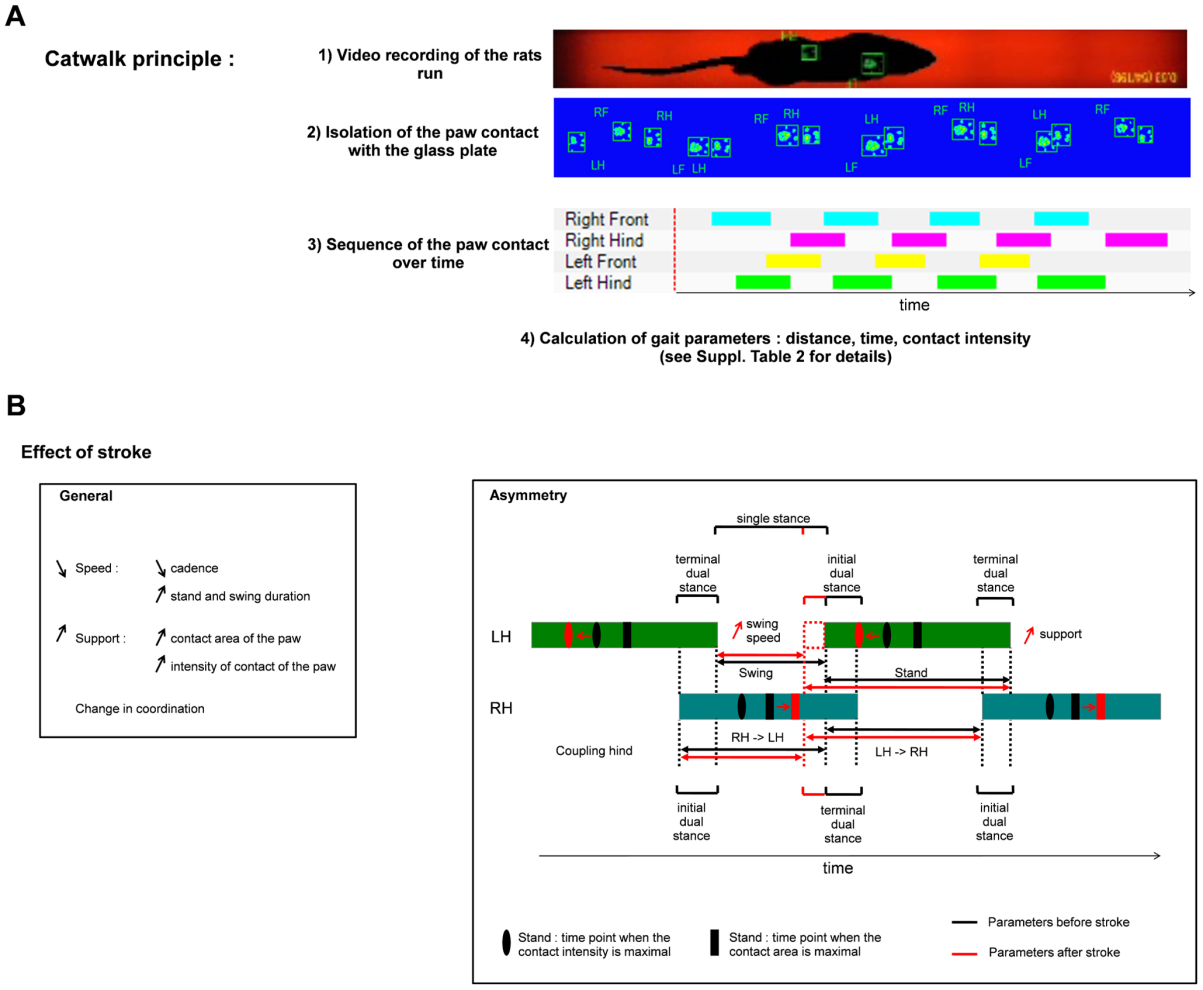
Effect of stroke on gait. Presentation of gait parameters and the changes induced by PT. (A) Principle of the Catwalk. Rats run on a glass plate illuminated from the side. When the paws press the plate, light is diffracted and captured by a camera below the glass plate. The calculation of the gait parameters require the following steps: 1) the runs are recorded, 2) the light spots are associated with their respective paws, 3) the sequence of paw contact with the glass plate over time is determined and 4) the different gait parameters are calculated. (B) Stroke induces changes in different gait parameters. Here are summarized the general effect of stroke on gait two days after stroke. (C) Stroke also induces an asymmetry between left and right side in some gait parameters. The colored boxes represent time fractions when the paws are in contact with the surface of the glass plate. The black lines represent the values before stroke and the red lines the direction as well as increase or decrease of the values at 2 days after stroke (LH: left hind limb, RH: right hind limb). For details see Table S3.

Gait reflects the relative position of the body as seen in limb movement and positioning during locomotion over solid ground. Studies of gait following stroke also provide information on limb coordination and compensatory movements due to deficits in sensori-motor function. The Catwalk software provides 32 parameters describing the gait of the individual rat and thereby generates a large amount of data (Table S2). We analyzed gait at 2 days after PT in all animals prior to moving them into different housing conditions. Hence, at two days after stroke, animals performed with a reduced average speed and exhibited shifts in paw support and coordination (Table S3). Moreover, stroke induced asymmetry in the gait, especially in the hind paws (Table S4). The animals suffered from a lack of support on the paretic side and this results in an increased support on the non-paretic side (larger print area) but also on an accelerated swing, where the weight of the animal was on the paretic paw (shorter swing period, higher swing speed). This lack of support on the paretic paw, led to a longer support time on the non-paretic side before lifting up the affected paw (longer terminal dual stance). Similar but less pronounced changes in the initial/terminal dual stance and in the coupling of front paw were also seen (Fig. 4). We analyzed if there was a difference in gait parameters between the STD and EE groups at 2 and 5 weeks of recovery. No differences between the STD and EE groups were found (Table S5).

### Enriched housing enhances sensori-motor function after lesions to motor cortex

#### Neuroscore

**Figure 5:**
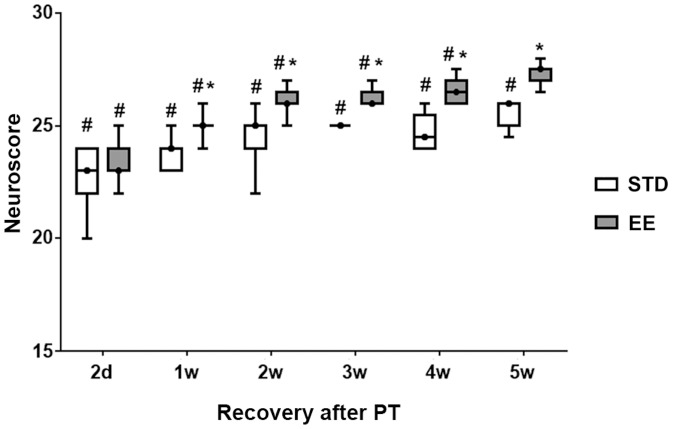
Neuroscore. The normal neuroscore before stroke is 28. Values at different times after PT in rats subsequently housed in standard (STD) and enriched environment (EE) are presented in box plot: median; 1^st^ quartile to 3^rd^ quartile, minimum and maximum value. (n = 7 for each housing condition) (*: p<0.05 when STD compared to EE; #: p<0.05 when STD and EE where compared to the respective values before stroke).

General neurological function was assessed using the 28-point neuroscore. On day 2 after stroke, the median score was 23 in the two groups destined subsequently to either STD or EE (Fig. 5). Hence, the deficit was similar among the experimental groups before initiating the enriched housing. Also, a rapid recovery of most behavioral parameters was evident at 2 days after stroke in this test. Among the 11 tests, only three tests were affected by the stroke at 2 days of recovery: the paw placement (see below), the grip strength and the fore paw reaching test. The animals housed in the STD group showed a persistent deficit up to the 5^th^ week, while the EE animals recovered almost completely at this time point.

#### Whisker-paw test

**Figure 6:**
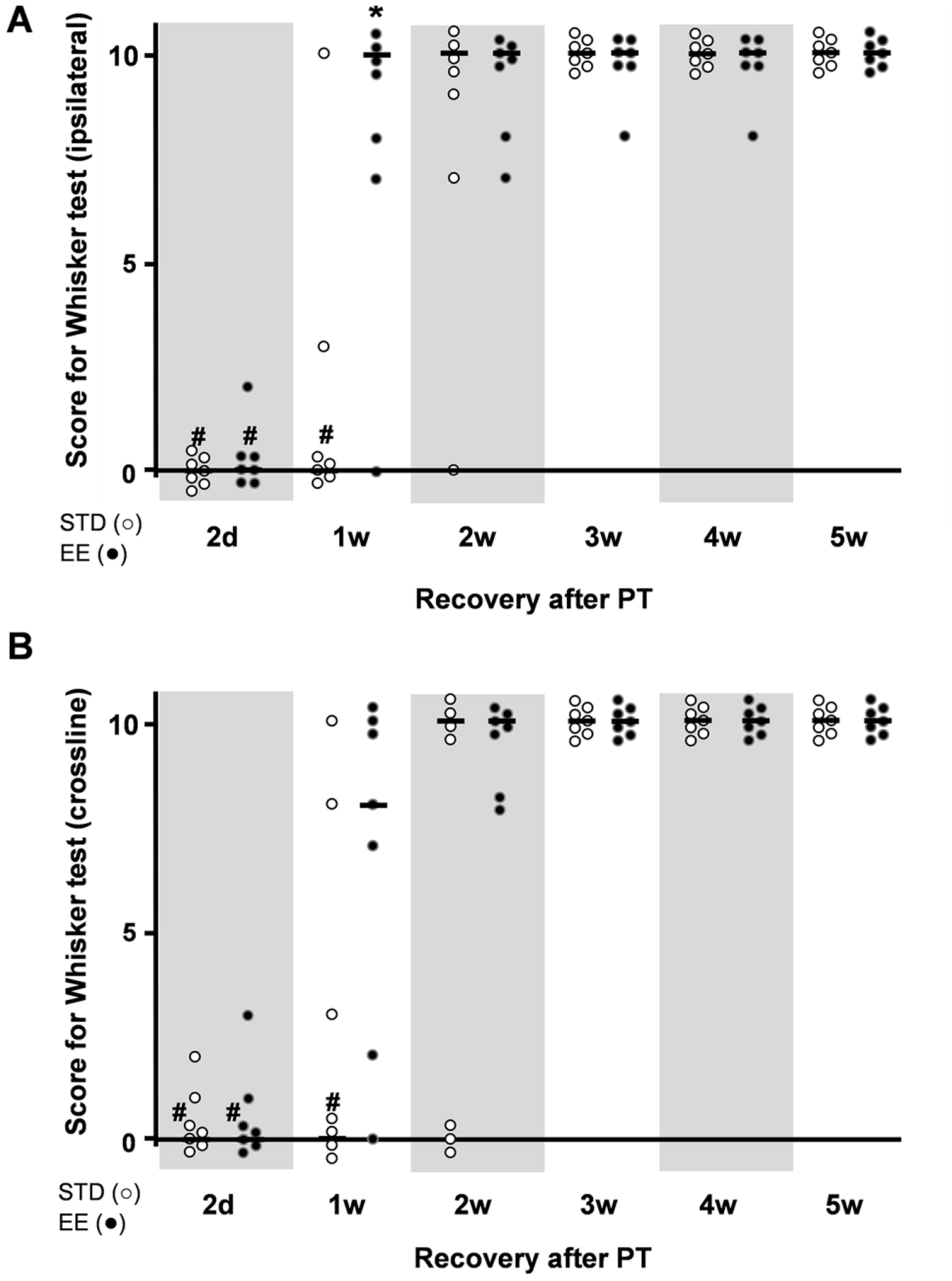
The whisker-paw test. (A) Ipsilateral whisker test, and (B) cross-midline whisker test of rats subjected to PT at various times of recovery. Animals were placed in a standard (STD) or an enriched (EE) environment at 2 days after stroke. The normal score before stroke for rats is 10. Each dot corresponds to a rat value and the black line corresponds to the median value for the group. (n = 7 for each housing condition) (*: p<0.05 when STD compared to EE; #: p<0.05 when STD and EE where compared to the respective values before stroke).

The WP test showed a marked deficit at 2 days after PT in both the STD and EE groups of rats (score 0), (Fig. 6). By 1 to 2 weeks of recovery and housing in enriched condition, the animals regained full score on this test (score 10). In the STD housing group, recovery was delayed by one week. Hence, by 3 weeks of recovery, these animals also regained the motor response to whisker stimulation, first in the ipsilateral WP stimulation paradigm and a week later in the cross-line stimulation.

#### Paw Placement test

**Figure 7:**
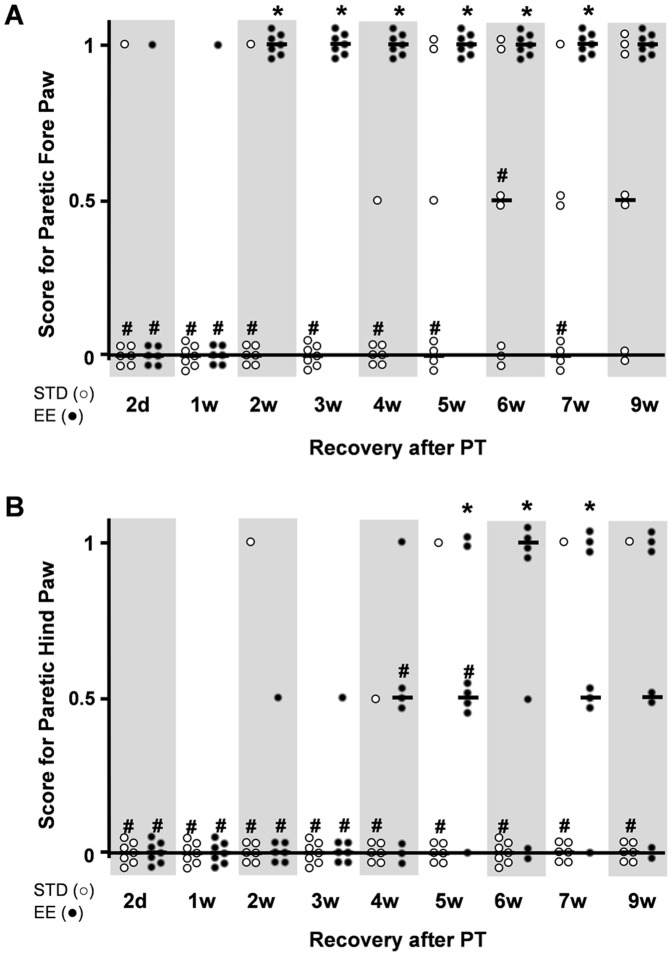
Paw placement test. Limb placing ability of rats subjected to PT under conditions that prevented visual and whisker/chin stimulation during testing. (A) Fore paw placement, and (B) hind paw placement. The normal score before stroke for rats is 1. Each dot corresponds to a rat value and the black line corresponds to the median value for the group. (n = 7 for each housing condition) (*: p<0.05 when STD compared to EE; #: p<0.05 when STD and EE where compared to the respective values before stroke; STD: standard environment, EE: enriched environment).

A remarkable difference in behavioral recovery between the housing groups was found in the PP test under conditions that prevented visual and whisker/chin stimulation during testing. In this test, the fore and hind paws on the side of the lesion were unaffected in both groups. The contralateral paws in the STD housed group recovered slowly, and by 9 weeks, 50% of the animals still displayed a deficit in right fore paw function and all but one animal had the lowest score in the hind paw function (Fig. 7). In contrast, all animals housed in the EE group displayed a rapid dynamic recovery. Fore paw placement recovered completely by 2 weeks (but not at 1 week) of recovery and the recovery persisted up to the 9-week time point. The hind paw placement recovered slower, but by 9 weeks 70% of the animals showed recovery, a time point when only 1 animal displayed recovery in the STD housed group.

### Enriched housing promotes a decrease in aggrecan-containing PNNs around PV/GABA neurons

To establish a structural correlate to the recovery of somatosensory function promoted by enriched housing, we investigated the integrity of PNNs. It has earlier been shown that the number of PNNs is affected by stroke, after both MCAO [49], [50] and PT [51], but, to our knowledge, there are no data linking these observations in the cerebrum with differential housing conditions and behavior. Since the true complexity of the extracellular matrix can only be appreciated if several protein epitopes are studied, we determined the stroke-induced modulation/distribution of perineuronal net-bearing cells by means of two antibodies recognizing different epitopes of aggrecan, the major constituent of PNNs in the adult rat brain [41].

**Figure 8:**
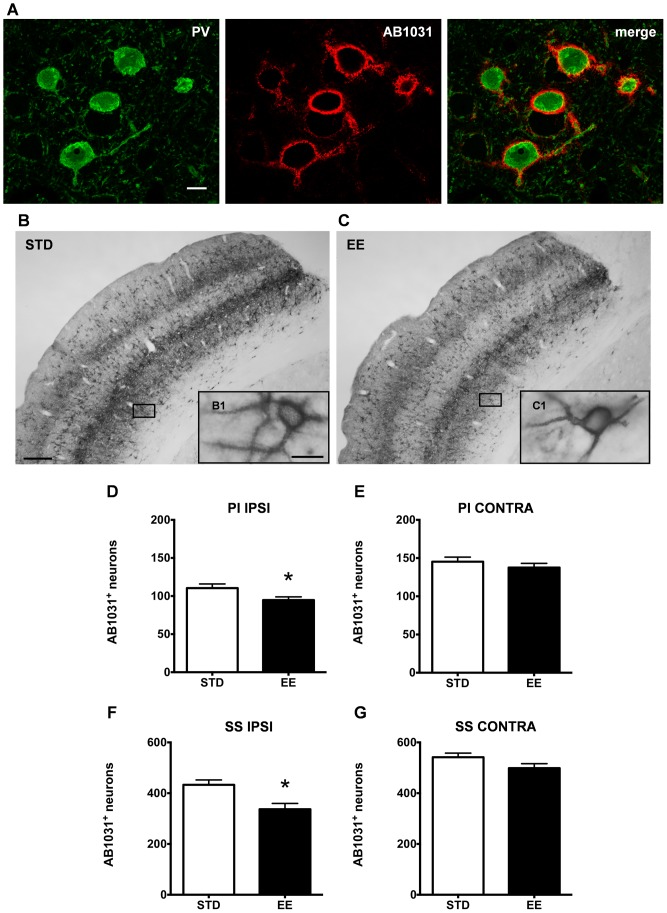
Environmental stimulation decreases the number of AB1031^+^ PV/GABA neurons in the rat cerebral cortex after PT. (A) Confocal images of parvalbumin expressing cells (green) enwrapped by AB1031^+^ PNNs (red) in the somatosensory cortex of a representative STD animal, scale bar 20 μm. (B and C) Representative bright-field micrographs of AB1031^+^ PNNs in the rat cerebral cortex ipsilateral to the lesion, scale bar 100 μm; (B1 and C1) higher magnification, scale bar 20 μm. AB1031 immunoreactivity in (B) STD and (C) EE conditions. Quantification of cortical neurons bearing AB1031^+^ PNNs in the peri-infarct cortex (D), the corresponding area contralateral to the lesion (E), the ipsilateral somatosensory cortex (F) and the contralateral somatosensory cortex (G). (n = 7 for each housing condition; 9 weeks after stroke; D with *p = 0.04; E with p = 0.3613; F with *p = 0.007; G with p = 0.0963; STD: standard environment, EE: enriched environment, PI: peri-infarct cortex, SS: somatosensory cortex, IPSI: ipsilateral, CONTRA: contralateral).

**Figure 9:**
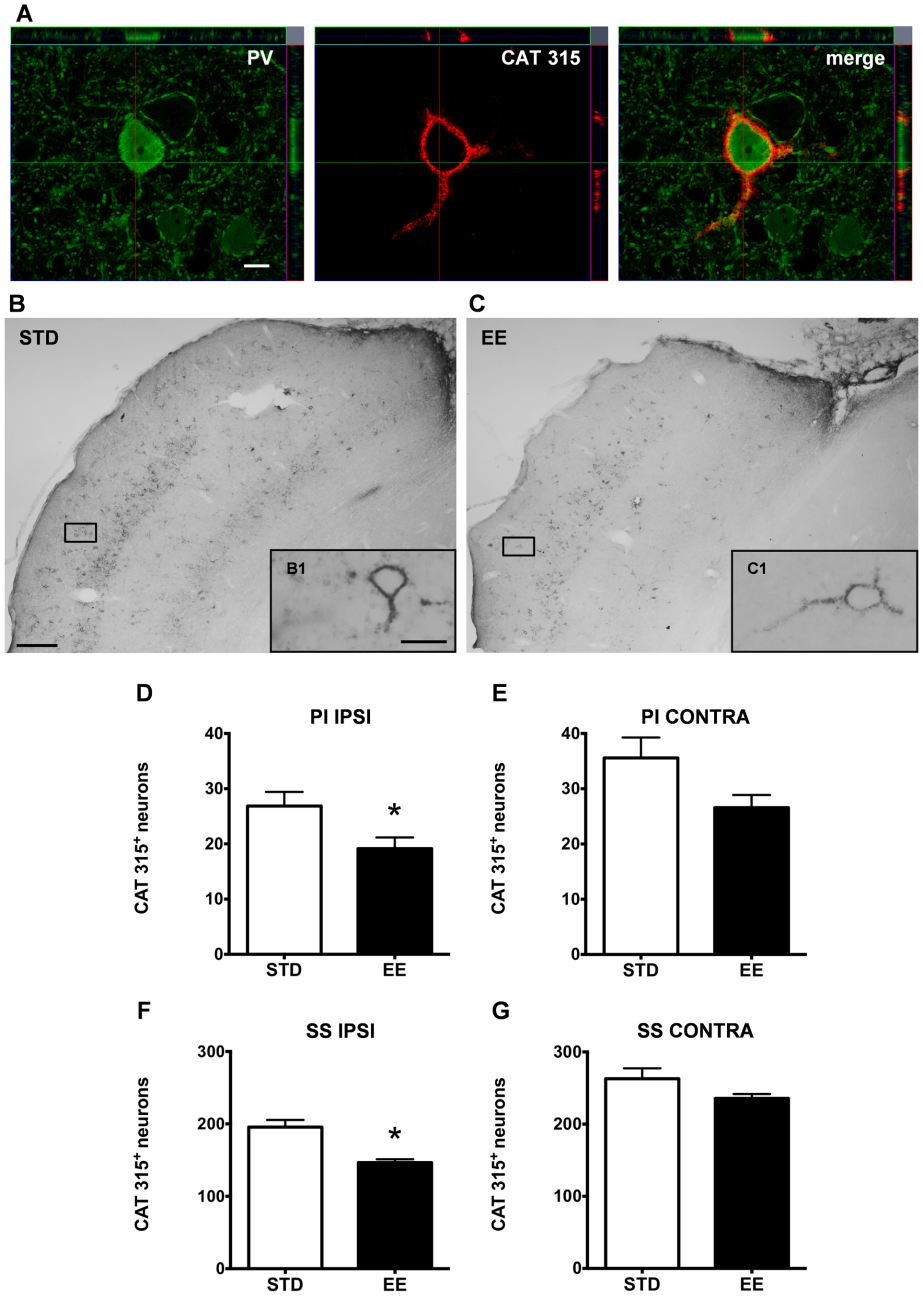
Enriched housing decreases the number of Cat-315^+^ PV/GABA neurons in the rat cerebral cortex after PT. (A) Confocal images of a parvalbumin (PV) expressing cell (green) enwrapped by a Cat-315^+^ PNN (red) in the somatosensory cortex of a representative STD animal; Z-stack demonstrates close proximity of the Cat-315 aggrecan antibody and PV, scale bar 20 μm. (B and C) Representative bright-field micrographs of Cat-315^+^ PNNs in the rat cerebral cortex ipsilateral to the lesion, scale bar 100 μm; (B1 and C1) higher magnification, scale bar 20 μm. Cat-315 immunoreactivity denotes a critical difference between (B) STD and (C) EE conditions. Quantification of cortical neurons bearing Cat-315^+^ PNNs in the peri-infarct cortex (D), the corresponding area contralateral to the lesion (E), the ipsilateral somatosensory cortex (F) and the contralateral somatosensory cortex (G). (n = 7 for each housing condition; 9 weeks after stroke; D with *p = 0.04; E with p = 0.06; F with *p = 0.0007; G with p = 0.1; STD: standard environment, EE: enriched environment, PI: peri-infarct cortex, SS: somatosensory cortex, IPSI: ipsilateral, CONTRA: contralateral).

Two antibodies were used to target different epitopes on the aggrecan molecule: AB1031 [60], [61], which recognizes an epitope within the central protein domain of aggrecan at chondroitin sulfate glycosaminoglycan binding sites, and Cat-315, which labels a glycan moiety of aggrecan in the adult [41], [43], [62]. In the following, we will refer to the AB1031 immunopositive and Cat-315 immunopositive cells as AB1031^+^ and Cat-315^+^ respectively. We examined the number of AB1031^+^ and Cat-315^+^ PNNs in the peri-infarct and somatosensory cortex of both hemispheres of rats kept in either housing condition after PT (Fig. 8, 9).

The aggrecan-positive PNNs surround neurons containing the calcium-binding protein parvalbumin [44], a widely accepted marker for GABAergic interneurons. We confirmed this notion with a double fluorescent staining for PV and both aggrecan antibodies (Fig. 8A, 9A), where we observed that the majority of PV^+^ neurons were also positive for either AB1031 or Cat-315 markers. We also confirmed the localization of PNN^+^ neurons in layers II, III, IV, V and VI (AB1031 staining, Fig. 8B, C) and in layers IV and VI (Cat-315 staining, Fig. 9B, C) in both the STD and EE groups.

In the injured hemisphere of animals housed in an EE, we found a significantly 13% reduction in the number of AB1031^+^ PNNs in the peri-infarct cortex (95±4 cells) when compared with animals housed in STD conditions (110±5 cells; Fig. 8D). In the homotopic area of the contralateral hemisphere there was no significant differences between the two groups (Fig. 8E; STD: 145±6 cells and EE: 138±5 cells). A significant 22% reduction in the number of AB1031^+^ PNNs was observed in the ipsilateral somatosensory cortex of the EE animals (433±19 cells) when compared to STD housing conditions (337±23 cells; Fig. 8F) while there was no difference in the contralateral hemisphere (Fig. 8G; STD: 542±16 cells and EE: 499±17 cells).

With regard to the Cat-315^+^ PNNs, we found a significant 30% decrease in the number of Cat-315^+^ PNNs (19±2 cells) in the peri-infarct area of EE animals (Fig. 9D) compared to the STD housed group (27±3 cells). In the homotopic area of the contralateral hemisphere, enriched housing also appeared to affect the number of Cat-315^+^ PNNs although the changes were not statistically significant (35±4 cells and 26±2 cells in the STD and EE groups respectively, p = 0.06, Fig. 9E). This tendency was also seen throughout the somatosensory cortex, where a 25% decrease of Cat-315^+^ PNNs in the EE group (146±5 cells) compared to the STD group (195±10 cells) was found (Fig. 9F). Again, no statistical significance was found in the homotopic region of the contralateral hemisphere, where we counted 263±14 cells in the STD conditions and 235±6 cells in the EE conditions (Fig. 9G).

## Discussion

In the present investigation we show that housing rats in an EE following an ischemic lesion to the fore and hind limb motor areas, enhances the speed of neurological recovery, particularly the recovery of the marked and persistent loss of tactile/proprioceptive limb placing response. The recovery-enhancing effect of EE is not due to a decrease in infarct size and sparing of brain tissue, but correlates with a robust reduction of aggrecan-containing PNNs surrounding parvalbumin-containing GABAergic neurons in the ipsilateral surviving cortical tissue. In the following discussion we will focus on the enhancement of somatosensory functions by enriched housing, the possible underlying biological mechanisms and its clinical implications.

### A model for somatosensory neglect and dysfunction

The PT model of stroke is advantageous in studying long-term recovery mechanisms after experimental stroke, since the localization and size of the lesion can be controlled with little variability among animals. With this model, plasticity processes affecting various brain regions, functions and cellular elements, which range from small infarcts induced by single vessel PT occlusions [63] to large brain lesions involving partial or entire functional entities such as the somatosensory cortex [64], [65], can be studied. Hence, depending on the size and the localization of the lesion, variable degree and speed of recovery is observed. With PT stroke, deficits in laterality, sensory or motor function and learning have been reported using the cylinder test, adhesive removal test [66], beam walking test, beam balanced test, corner test [67], rotarod [68], gap crossing test [69], Catwalk [70], skilled reaching, horizontal ladder test [71], neurological severity score [72], Morris water maze [73], plantar test [74], spontaneous activity and grip strength [75].

We induced the PT lesion in the fore and hind limb M1 areas [76], [77] leaving the medial agranular cortex and the somatosensory cortex essentially intact [78]. This lesion caused a deficit in several sensori-motor tests, albeit to varying degree and persistency. We confirm a deficit in skilled reaching that persisted throughout the 9 weeks of recovery [58]. Gait was affected 2 days post stroke, with changes in coordination, speed of movement as well as asymmetry in the support of the paws. The sensori-motor response to whisker stimulation, that was lost at day 2 after stroke, recovered within 2-4 weeks. The most dramatic effect on behavioral outcome was the deficit in tactile/proprioceptive response when visual stimuli and whisker/snout contact were prevented, as reported earlier [55], [78]. Here we found that the hind limb response was persistently depressed, while the fore limb function partially recovered by the 9^th^ week of recovery. Evidently, the extensive lesion to M1 caused marked and persistent deficits in fine motor skills due to the damage of the cortico-spinal tract (CST) and loss of somatosensory functions with a variable degree of recovery. When visual and whisker/chin stimulations were prevented, the recovery of limb placement was markedly depressed (fore limb) or prevented (hind limb). Therefore, this cortical PT lesion appears to induce a specific somatosensory neglect [55], [78].

### Enriched housing reverses sensory neglect and dysfunction

Using the EE paradigm, it is possible to assess the recovery potential of various lost brain functions following brain damage such as stroke. We initiated enriched housing at 2 days after PT, a time when the infarct can be considered fully matured. Subsequent enriched housing for 9 weeks did not influence infarct size, in line with earlier studies using this model [71]. Improved performance during enriched housing must therefore be associated with recovery of neuronal functions, connectivity and post-stroke brain plasticity.

Over the 9-week period studied, the animals housed in the EE did not improve skilled reaching. This is in accord with earlier findings using PT, where enriched housing promoted use of compensatory movements while fine motor skills essentially did not improve [71]. Presumably, the PT lesion damages the CST to such extent that little tissue is left to become substrate for network remodeling and recovery over the 9-week period studied. Longer periods of recovery could involve activation of the contralateral motor cortex [29], but this is not likely in a lesion of the present size [79]. Also, different results were obtained after transient and permanent occlusion of the middle cerebral artery, where the CST is largely preserved and amenable to plasticity and recovery [80], [81].

In contrast to the limited enhancement of fine skilled motor recovery by enriched housing, the speed of recovery of the sensori-motor deficit seen in the whisker tests at 1–2 weeks of recovery was markedly enhanced. Likewise, the tactile proprioceptive response (limb placement) where visual and whisker cues are hidden, displays an even more striking recovery enhancement induced by enriched housing. The lost fore limb placement fully recovered after 12 days of enriched housing. The hind limb placement response that never recovered in STD housed animals clearly responded after enriched housing.

Hence, we observe a beneficial effect of the enriched housing when using tests of sensory abilities and proprioception but no marked effect when using tests of skilled motor functions or locomotion. This suggests that the STD housed animals fail to integrate sensory stimuli to trigger a motor response, particularly when visual and whisker stimuli are absent. The EE, stimulating the somatosensory network involved in the sensori-motor response, evidently promotes cellular and network plasticity and provides improved recovery. The peri-infarct area has been proposed to constitute a growth permissive region that promotes axonal outgrowth and synaptogenesis [82]. The recovery of tactile sensation and proprioception could be envisaged to involve sensori-motor cortex also remote from the lesion. We therefore investigated changes in the integrity of PNNs, known to be affected by enriched housing [83], [84].

### Enriched housing enhances reduction of aggrecan-containing PNNs after experimental stroke

We show here for the first time that the multisensory stimulation provided by enriched housing after experimental stroke reduces the number of aggrecan-containing PNNs in the peri-lesional area, as well as in the ipsilateral somatosensory cortex remote to the lesion.

CSPGs are generally up-regulated in the peri-infarct regions after experimental stroke as part of the glial scar formed to contain the infarct [50], [85]. However, in areas remote from the ischemic lesion, CSPGs are reduced [49], contributing to the formation of a growth-permissive region surrounding the infarct after injury [86]. Our data suggest that the multisensory stimulation provided by enriched housing further expands this window of experience-dependent plasticity in space and time after stroke, including more remote somatosensory cortical areas, or even the contralateral hemisphere, several months after stroke. This is in line with recent reports that degradation of CSPGs with chondroitinase ABC improves functional recovery after experimental stroke [87]–[89].

We also found that the Cat-315^+^ PNNs, staining a glycan moiety of aggrecan, are more reduced than the AB1031^+^ PNNs, staining the aggrecan protein backbone. This would indicate that a subset of neurons (Cat-315^+^) is particularly sensitive to enriched housing conditions after experimental stroke [62]. Earlier studies have demonstrated that sensory deprivation reduces the number of Cat-315^+^ PNNs [43], [90]. Still, we do not know if the reduction of aggrecan-containing PNNs in our study is due to enhanced degradation or a reduced synthesis of aggrecan after experimental stroke. The Cat-315^+^ neurons enwrap mainly parvalbumin-containing GABAergic neurons [43], [44], of crucial importance for processing of sensori-information [91] and hence also of importance in functional recovery after stroke. Indeed, the wide spread reduction of aggrecan-containing PNNs in the somatosensory cortex suggests changes in sensory processing enhanced by enriched housing and involving neural networks remote from the lesion.

### Conclusions

The PT stroke lesion to fore and hind paw regions of the rat brain provides a tool and model to study the mechanisms of somatosensory dysfunction and recovery after stroke. We propose that multisensory stimulation provided by enriched housing reduces the density of PNNs surrounding PV/GABA neurons, improving cortical plasticity and sensori-information processing. Elucidating the mechanisms and pharmacology of PNN turnover after experimental stroke may provide new therapies supporting rehabilitation.

## Supporting Information

Table S1 Animal data. Parameters of the rats subjected to PT with subsequent housing in STD or EE.
(TIF)

Table S2 Explanation of the Catwalk parameters. (a.u.: arbitrary units).
(TIF)

Table S3 Gait parameters affected by stroke. The pre-stroke values and the values 2 days after stroke are presented (n = 14) as well as the percentage of variation between the time points, the p-value of the Student's t-test (pre *vs*. 2d), the Pearson's correlation coefficient with the speed and weight with the respective associated p-values. (RF: right front, RH: right hind, LF: left front, LH: left hind, a.u.: arbitrary unit).
(TIF)

Table S4 Asymmetry in parameters of gait induced by stroke. Gait parameters where differences were seen between paretic and non-paretic side at 2 days after stroke. The pre-stroke values and the values 2 days after stroke are presented (n = 14) as well as the percentage of variation between the two side, the respective p-value of the Student's t-test (L *vs*. R) for both time and the p-value of the Student's t-test on the difference (Right-Left) before and 2d after stroke. (F: front, H: hind, L: left, R: right).
(TIF)

Table S5 Recovery in parameters of the gait affected by stroke. Gait parameter values affected by stroke (general) and the difference between paretic and non-paretic side induced by stroke (asymmetry) at 2 and 5 weeks in rats housed in either STD or EE. (RF: right front, RH: right hind, LF: left front, LH: left hind, F: front, H: hind).
(TIF)
