# Supplementary figures and images for: Enriched Housing Enhances Recovery of Limb Placement Ability and Reduces Aggrecan-Containing Perineuronal Nets in the Rat Somatosensory Cortex after Experimental Stroke
Source: PLoS One. 2014 Mar 24;9(3):e93121. doi: 10.1371/journal.pone.0093121 (PMC3963994; doi:10.1371/journal.pone.0093121)

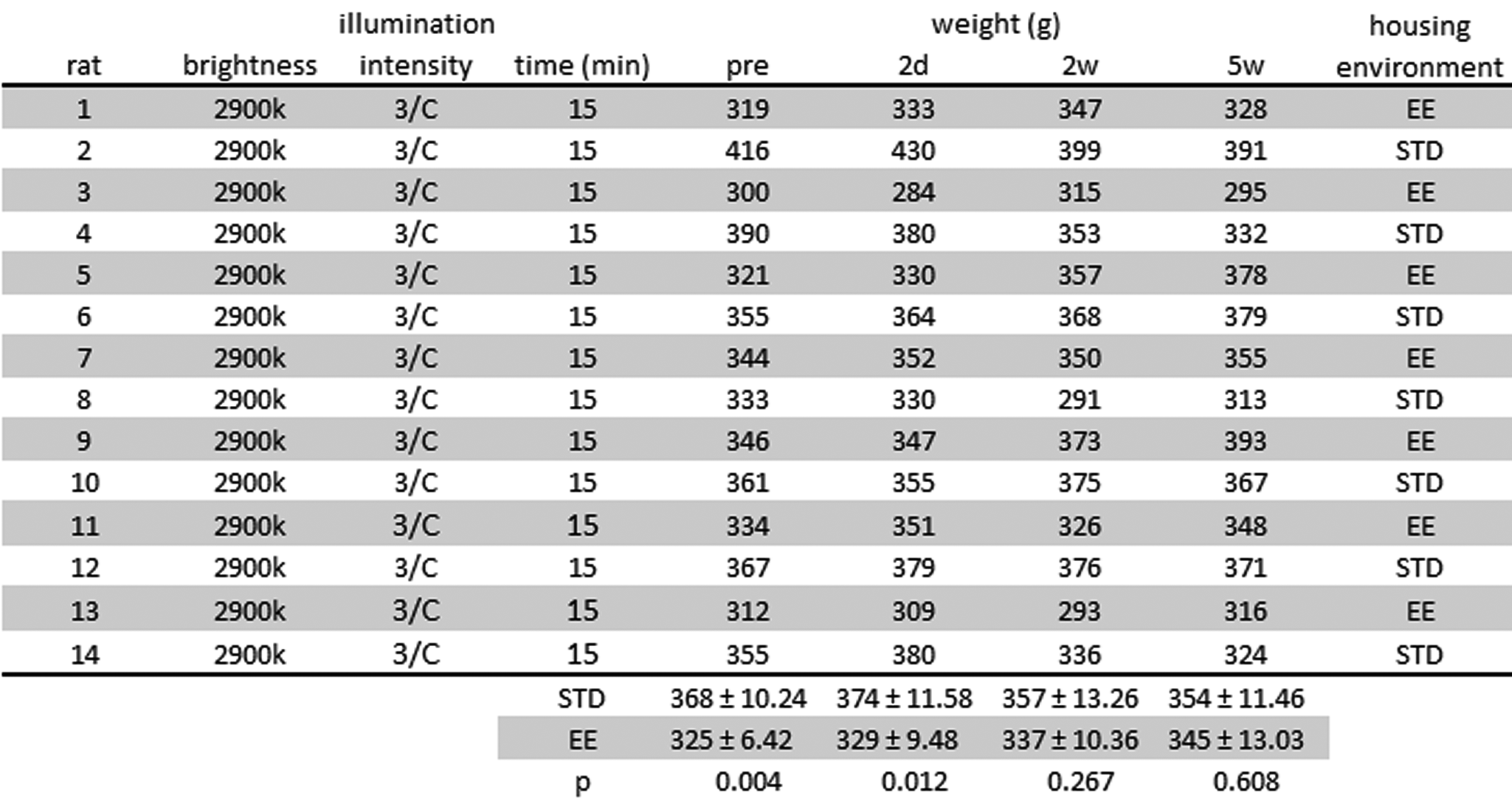

Supplement: Table S1 — Animal data. Parameters of the rats subjected to PT with subsequent housing in STD or EE. (TIF) [file pone.0093121.s001.tif]

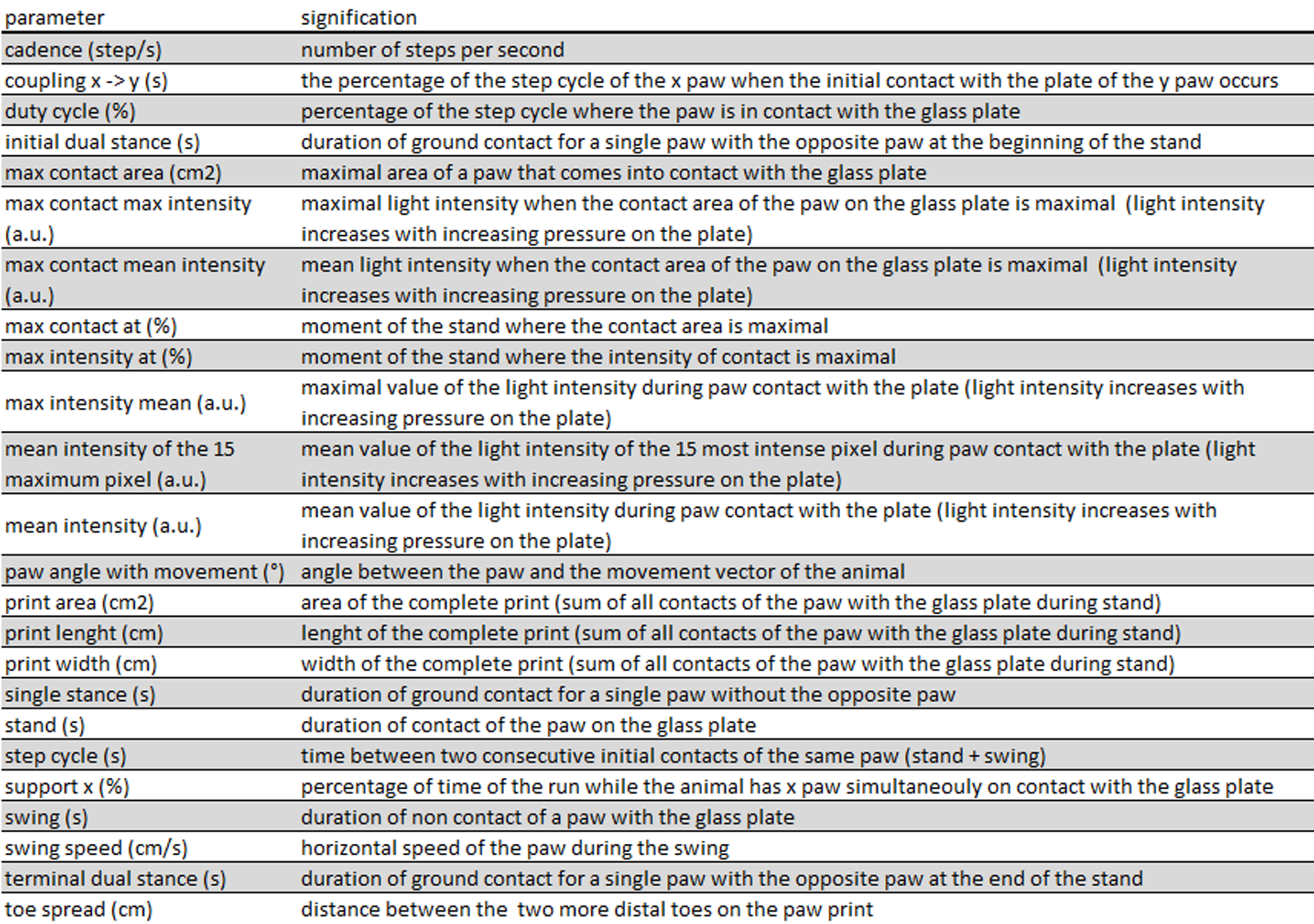

Supplement: Table S2 — Explanation of the Catwalk parameters. (a.u.: arbitrary units). (TIF) [file pone.0093121.s002.tif]

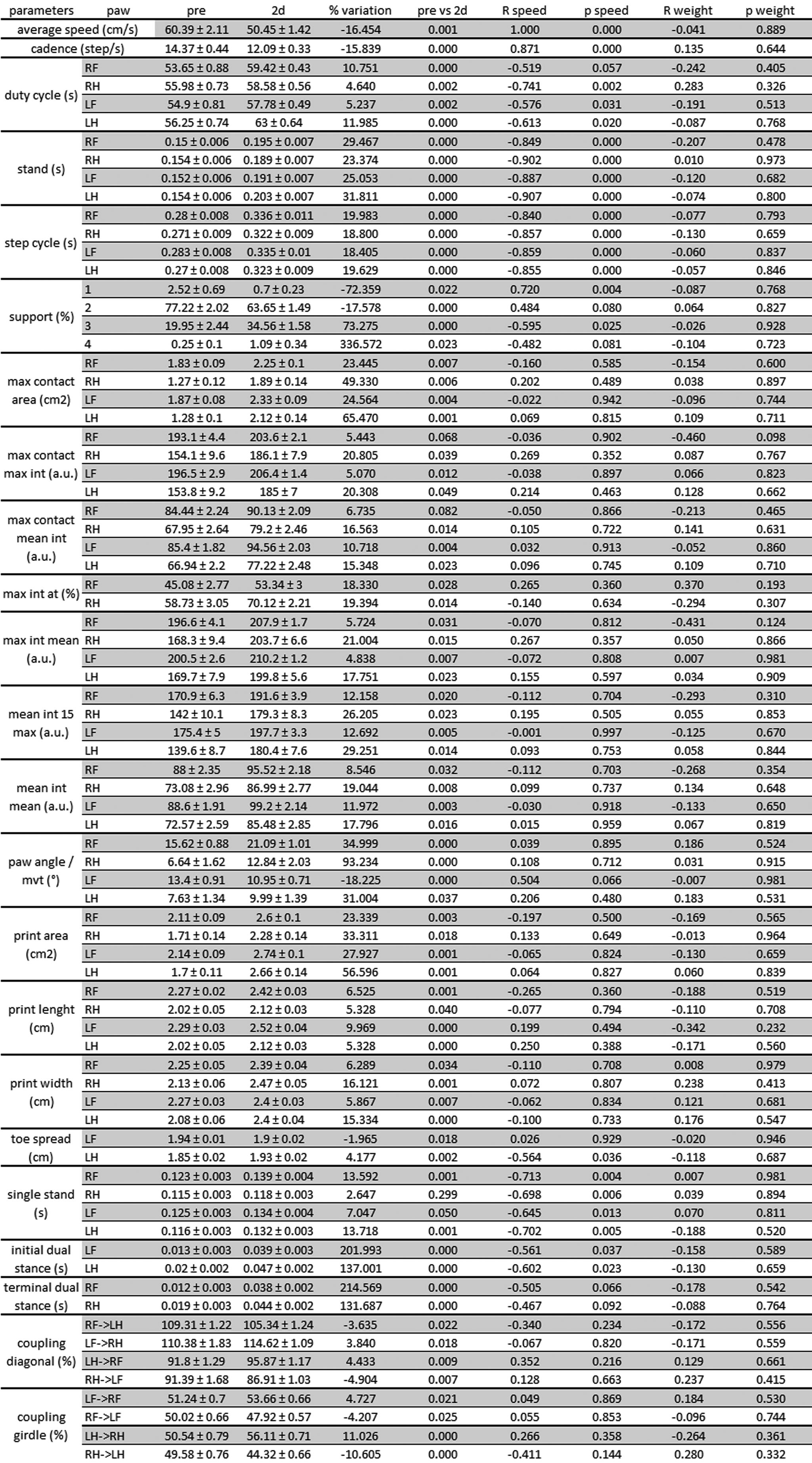

Supplement: Table S3 — Gait parameters affected by stroke. The pre-stroke values and the values 2 days after stroke are presented (n = 14) as well as the percentage of variation between the time points, the p-value of the Student's t-test (pre vs. 2d), the Pearson's correlation coefficient with the speed and weight with the respective associated p-values. (RF: right front, RH: right hind, LF: left front, LH: left hind, a.u.: arbitrary unit). (TIF) [file pone.0093121.s003.tif]

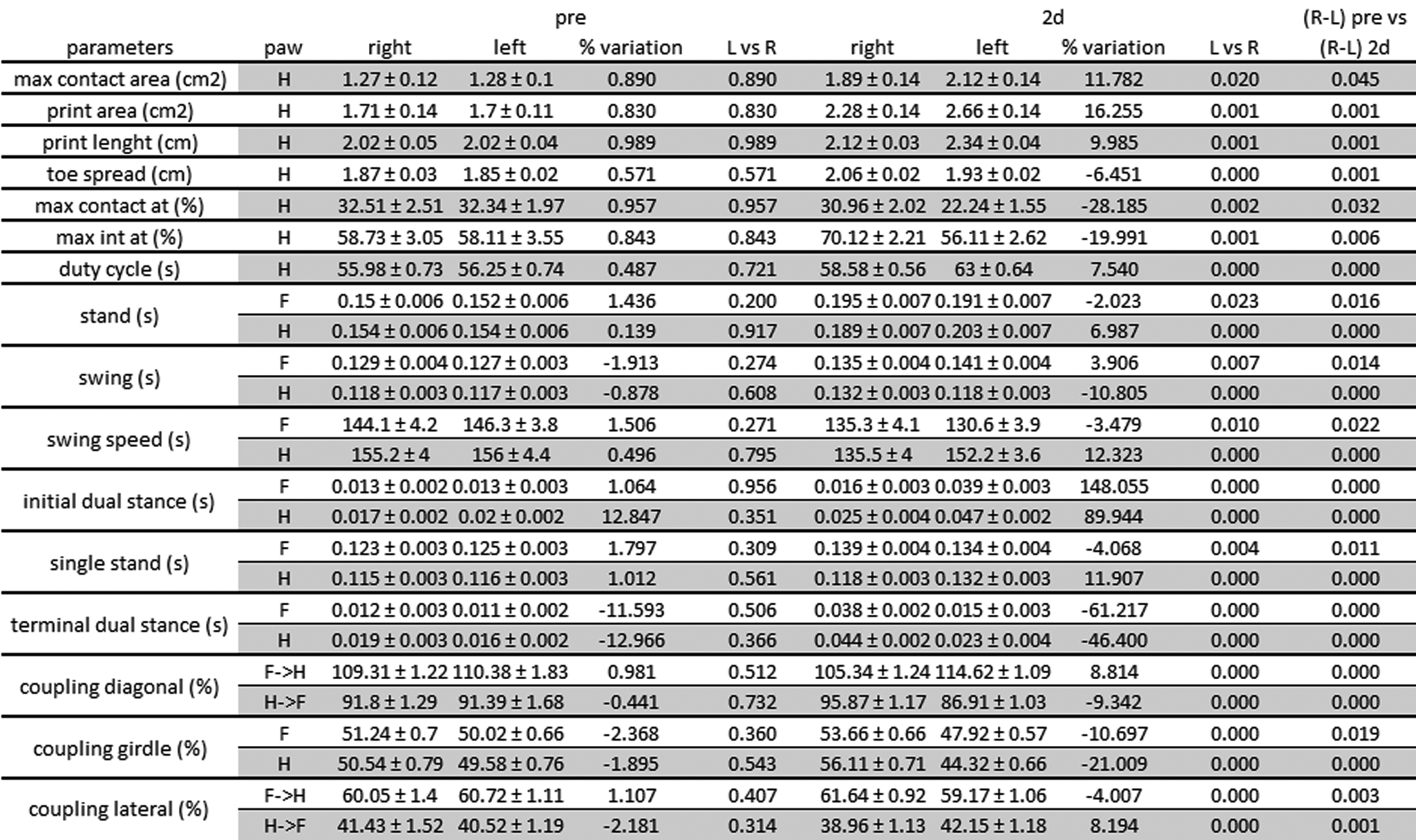

Supplement: Table S4 — Asymmetry in parameters of gait induced by stroke. Gait parameters where differences were seen between paretic and non-paretic side at 2 days after stroke. The pre-stroke values and the values 2 days after stroke are presented (n = 14) as well as the percentage of variation between the two side, the respective p-value of the Student's t-test (L vs. R) for both time and the p-value of the Student's t-test on the difference (Right-Left) before and 2d after stroke. (F: front, H: hind, L: left, R: right). (TIF) [file pone.0093121.s004.tif]

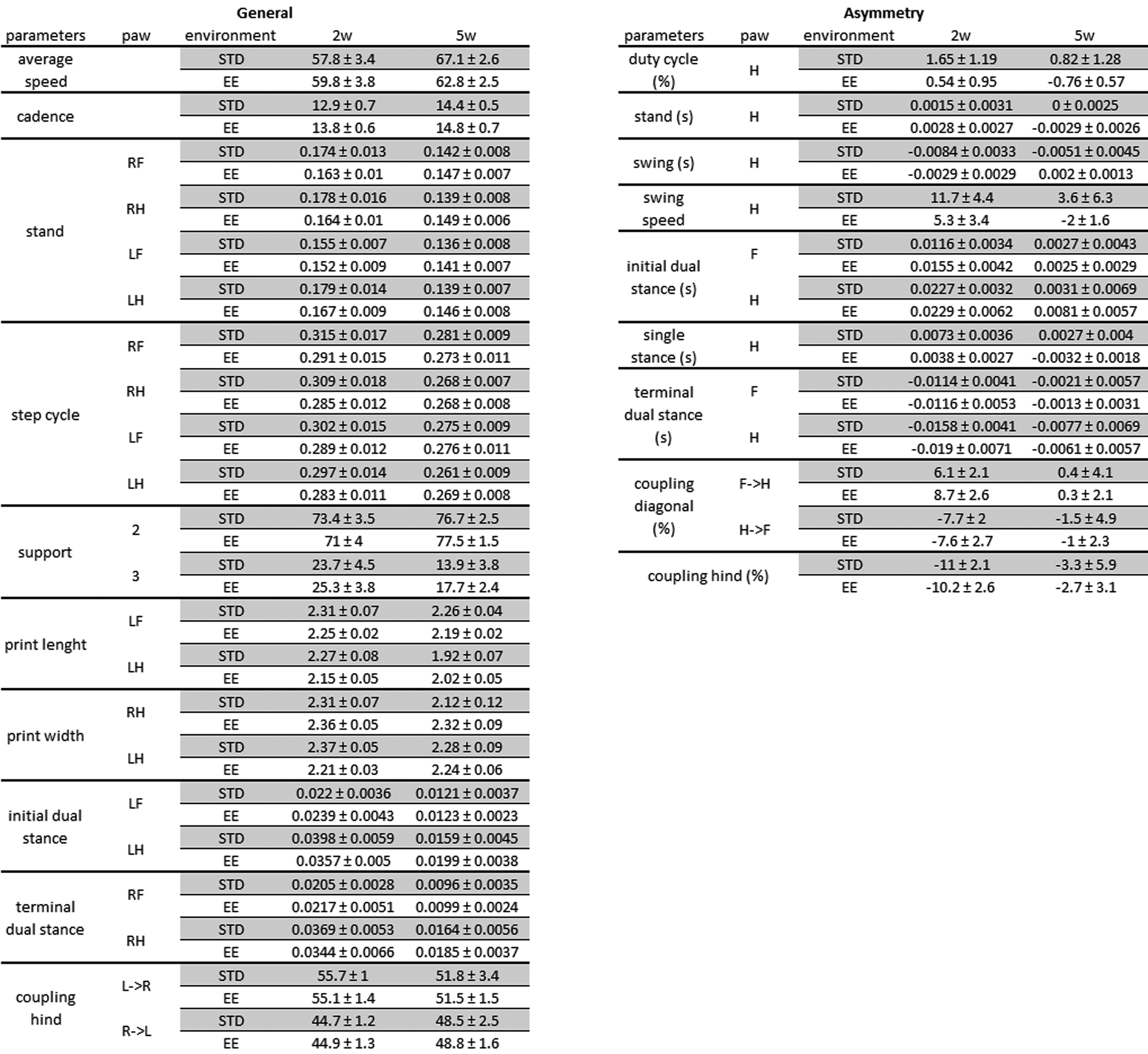

Supplement: Table S5 — Recovery in parameters of the gait affected by stroke. Gait parameter values affected by stroke (general) and the difference between paretic and non-paretic side induced by stroke (asymmetry) at 2 and 5 weeks in rats housed in either STD or EE. (RF: right front, RH: right hind, LF: left front, LH: left hind, F: front, H: hind). (TIF) [file pone.0093121.s005.tif]
